# Understanding of chest pain in microvascular disease proved by cardiac magnetic resonance image (UMPIRE): study protocol for a randomized controlled trial

**DOI:** 10.1186/1745-6215-15-333

**Published:** 2014-08-26

**Authors:** Sung-Ji Park, Jin Joo Park, Dong-Ju Choi, Eun Ju Chun, Sang Il Choi, Sung Mok Kim, Shin Yi Jang, Soyeon Ahn, Yeon Hyeon Choe

**Affiliations:** Cardiovascular Imaging Center, Heart, Vascular, Stroke Institute, Samsung Medical Center, Sungkyunkwan University School of Medicine, #50 Irwon-dong, Gangnam-gu, Seoul, 135-710 Korea; Cardiovascular Center, Seoul National University Bundang Hospital, Internal Medicine, College of Medicine, Seoul National University, Gumiro 166, Bundang-gu, Seongnam-si, Gyeonggi-do, 463-707 Republic of Korea; Department of Radiology, College of Medicine, Seoul National University Bundang Hospital, Gumiro 166, Bundang-gu, Seongnam-si, Gyeonggi-do, 463-707 Republic of Korea; Medical Research Collaborating Center, Seoul National University Bundang Hospital, Seongnam, Gumiro 166, Bundang-gu, Seongnam-si, Gyeonggi-do, 463-707 Republic of Korea

**Keywords:** Microvascular angina, PDE-5 inhibitors, Cardiac MRI

## Abstract

**Background:**

Microvascular angina (MVA) is characterized by anginal chest pain, an abnormal stress test, and normal coronary arteries on coronary angiography. Although the exact pathogenesis remains unclear, endothelial dysfunction is a contributing factor. To date, there exists no specific therapy for this disease. Phosphodiesterase-5 inhibitor improves the endothelial function and subsequently microvascular circulation. The aim of this study is to identify whether udenafil offers benefits in the treatment of MVA in female patients, who have a perfusion defect in their cardiac magnetic resonance image (CMR), but normal coronary arteries.

**Methods/Design:**

The ‘Understanding of Chest Pain in Microvascular Disease Proved by Cardiac Magnetic Resonance Image: (UMPIRE)’ trial is a multicenter, prospective, randomized, placebo controlled trial, designed to evaluate the effect of udenafil on myocardial ischemia and symptoms in female patients with MVA. The myocardial ischemia will be quantified by myocardial stress perfusion defect in CMR. A total of 80 patients with proven perfusion defect in adenosine-stress CMR will be randomly assigned to either the udenafil treatment group (daily dose of 100 mg) or the placebo group for three months. The primary endpoint is >25% improvement in perfusion defect size in adenosine-stress CMR from baseline. The secondary endpoints include <25% improvement in perfusion defect size, chest pain frequency, ST depression in stress test, Duke score in stress test, quality of life (QoL) assessment by SF-36 questionnaire, sexual dysfunction assessment by BISF-W (Brief Index of Sexual Functioning for Women) self-assessment questionnaire, and biomarkers for endothelial function.

**Discussion:**

The UMPIRE trial is the first randomized controlled trial to evaluate the efficacy of udenafil in female MVA patients. If udenafil demonstrates cardioprotective effects, it may provide a novel therapeutic option to reduce myocardial ischemia and improve cardiac function in female MVA patients.

**Trial registration:**

Clinical Trials.gov: NCT01769482 (registered on 20 November 2012).

## Background

Microvascular angina (MVA), also known as cardiac syndrome X, is characterized by anginal chest pain, an abnormal stress test indicative of myocardial ischemia, and normal coronary arteries on angiography. The condition is not as benign as originally reported. While patients with stable MVA have an excellent prognosis [1], unstable MVA patients presenting with acute coronary syndrome have an increased risk of death or myocardial infarction [2]. Women are more affected than men. The current therapeutic options for MVA are limited.

An impairment of endothelium-dependent vasodilation due to reduced nitric oxide (NO) release mainly accounts for the proposed mechanisms of MVA [3]. NO availability plays a critical role in the regulation of microvascular endothelial functions and the arterial structure [4–6]. Many of the biological actions of NO are mediated by 3’5’-guanosine monophosphate (cGMP), which is rapidly degraded by cGMP phosphodiesterase (PDE). Recently, phosphodiesterase-5-inhibitor (PDEi) has been reported to improve the endothelial function in animal and clinical studies [7–10]. PDE inhibitors improve cardiac performance in patients with heart failure [11] and reduce infarct size and attenuate cardiac dysfunction after ischemia/reperfusion injury or permanent coronary artery occlusion [12]. While short-term cardiovascular effects of PDE inhibitors have been studied, the effects of chronic PDEi administration are not well known, especially in women with MVA.

In the ‘Understanding of Chest Pain in Microvascular Disease Proved by Cardiac Magnetic Resonance Image: (UMPIRE)’ study, we tested whether udenafil offers benefits in the treatment of female MVA patients who have a documented perfusion defect in CMR.

## Methods/Design

### Overview

**Figure 1:**
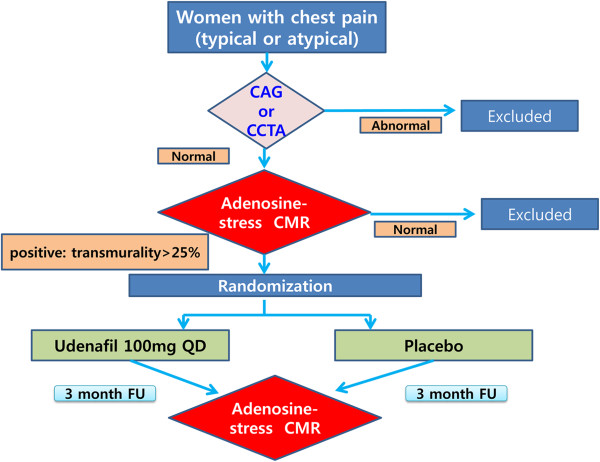
**Study flowchart.** CAG; coronary angiography, CTCA; coronary CT angiography, CMR*;* Cardiac magnetic resonance image.

This trial will be a multicenter, prospective, randomized, double-blind, placebo controlled trial to demonstrate the superiority of the therapeutic benefit of low-dose udenafil over a placebo in the treatment of female MVA patients. The working hypothesis is ‘udenafil improves vascular function and, consequently, alleviates ischemia in women with MVA who have a confirmed perfusion defect in CMR and normal coronary arteries on coronary CT angiography (CCTA) or coronary angiography’.The protocol of the trial has been registered with Clinicaltrials.gov (registration number: NCT 01769482) and a brief flowchart of the whole study is summarized in Figure 1.

### Endpoints

The primary endpoint is a >25% improvement from baseline in perfusion defect size in adenosine-stress CMR after three months of udenafil treatment. The secondary endpoints include a <25% improvement in perfusion defect size, chest pain, quality of life, and sexual functions after three months of udenafil treatment. Detailed endpoints are listed in Table 1.

**Table 1:** **Endpoints**

	Endpoint details
Primary endpoint	>25% improvement in perfusion defect size in adenosine-stress CMR from baseline to three months after udenafil treatment
Secondary endpoints	<25% improvement in perfusion defect size in adenosine-stress CMR from baseline to three months after udenafil treatment
	Decrement of frequency of chest pain
	Improvement of ST-depression in stress test
	Improvement of Duke score in stress test
	Improvement of QoL assessment by SF-36 questionnaire
	Improvement of sexual dysfunction assessment by BISF-W self-questionnaire
	Improvement of biomarkers for endothelial function

CMR*;* Cardiac magnetic resonance image, QoL; Quality of life.

### Patient population

Patients who are at least 18 years of age, have chest pain and positive adenosine-stress CMR, and normal coronary artery on CCTA or coronary angiography will be included in this study. A positive adenosine-stress CMR is defined as a perfusion defect of >25% of transmurality. Two high-volume centers in the Republic of Korea will participate and enroll eligible patients. The following patients are excluded from the study: patients with contraindication to MR contrast media or MR imaging, any heart rhythm other than sinus rhythm, congestive heart failure, chronic kidney disease, contraindication to PDEi, or uncontrolled diabetes mellitus. The detailed inclusion and exclusion criteria are summarized in Table 2.

**Table 2:** **Inclusion and exclusion criteria**

**Inclusion criteria**	
1	MVA patients with typical symptom and positive adenosine-stress CMR and with normal coronary artery in CCTA or coronary angiography.
2	Definition of positive adenosine-stress CMR: perfusion defect >25% of transmurality (by two radiologists based on visual assessment and qualitative assessment in core-lab)
3	Gender: female
4	Age: 18 to 80-years-old
**Exclusion criteria**	
1	Patient has a contraindication to CMR contrast media or CMR imaging
2	LVEF <50%
3	Any heart rhythm abnormality other than sinus rhythm
4	Valvular heart disease with more than moderate degree
5	Renal failure
6	Congestive heart failure
7	Myocardial infraction
8	Myocarditis
9	Congenital heart disease
10	Pericarditis
11	Variant angina (positive provocation test with ergonovine or acetylcholine)
12	GERD (conformed by esophagogastroduodenoscopy)
13	Pregnant women, suspected pregnant women or lactating women
14	QT prolongation syndrome or taking drugs that prolong the QT interval:
	- Antiarrhythmics class IA; quinidine, procainamide
	- Antiarrhythmics class III; amiodarone, sotalol
15	Pre-analytical within 30 days of screening in a clinical trial that may affect the influence of udenafil:
	- Other PDE-5 inhibitors (for example sildenafil, tadalafil)
	- Nitrates/NO donor (for example nitroglycerin, isosorbide mononitrate, isosorbide dinitrate, amyl nitrate/nitrite, sodium nitroprusside, and nicorandil)
16	Pre-analytical within seven days of screening in a clinical trial that may affect the metabolism of udenafil:
	- Antibacterials (for example erythromycin)
	- Antifungals (for example itraconazole, ketoconazole)
	- Antivirals (for example ritonavir, saquinavir, amprenavir, indinavir, nelfinavir)
	- Cimetidine
	- Grapefruit juice
17	Allergy or sensitivity with PDE-5 inhibitors

CCTA; Coronary CT angiography, CMR; Cardiac magnetic resonance image, GERD; Gastroesophageal reflux disorder, LVEF; Left ventricular ejection fraction, MVA; Microvascular angina, NO; Nitric oxide, PDE-5; Phosphodiesterase-5.

If all of the inclusion criteria are met and none of the exclusion criteria apply, the patient will be required to provide written informed consent, as required by the institutional review board (Samsung Medical Center Institutional Review Board, Seoul National University Bundang Hospital Institutional Review Board) in accordance with the Declaration of Helsinki.

### Randomization and interventions

After the patients are enrolled in the study, the patients will be randomized to the udenafil treatment group or the placebo group by a web-based computerized program which will be independently managed at T&W software (Three and Water software, Seoul, Republic of Korea). The flowchart of the study design is shown in Figure 1.

### Udenafil treatment protocol

After random assignment, patients in the udenafil treatment group will receive 100 mg udenafil daily (Dong-A Pharmaceutical, Seoul, Republic of Korea) for three months. Since headache and facial flushing are common side effects of udenafil, the dose of study medication will be reduced by 50% if severe headache develops. In patients with serious side effects the study medication will be stopped.

### Cardiac magnetic resonance imaging (CMR)

#### CMR imaging

##### Cine imaging

**Figure 2:**
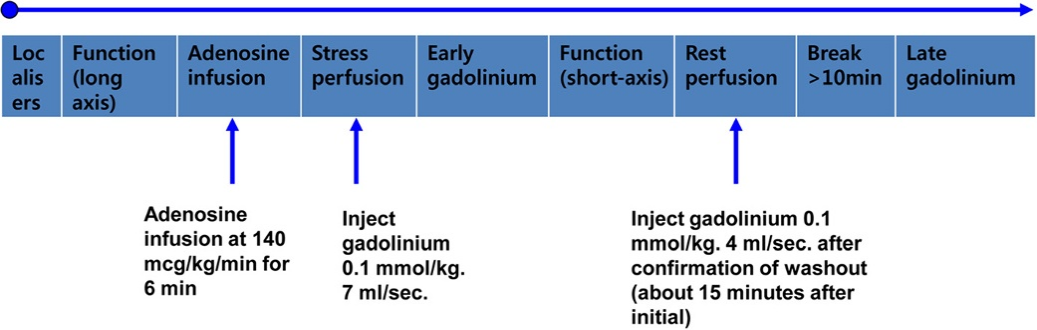
**Adenosine-stress CMR protocol.** Following image localizers, ventricular long cine images will be acquired. Adenosine (140 mcg/kg/min) will be infused for 6 minutes before stress perfusion imaging at the time of gadolinium injection. Early gadolinium imaging will be followed by short axis cine and then rest perfusion imaging. Late gadolinium enhancement images to evaluate the scar and patient viability will conclude the scan. Study duration will typically be 35 to 40 minutes.

All CMR studies will be performed using a 1.5 T MRI scanner (Siemens Avanto, Erlangen, Germany or Intera CV release 10; Philips Medical Systems, Best, Netherlands) with a phased array cardiac coil. Hemodynamic parameters, oxygen saturation, and lead vector ECG (Electrocardiogram) rhythm will be continuously monitored throughout the whole CMR exam. Following image localizers, ventricular long and short axis cine images will be acquired in end expiration with retrospectively ECG-gated steady-state free precession sequences (image matrix 128 × 128, read field of view (FOV) 340 mm, phase FOV 75 to 100% of the FOV read, echo time 1.12 ms, flip angle 70°) with slice thickness 8 mm and intersection gaps of 12 to 15 mm. The adenosine-stress CMR protocol is illustrated schematically in Figure 2.

##### Adenosine infusion protocol

Adenosine (Adenoscan™, Sanofi-Synthelabo, Berlin, Germany) will be infused at 140 μg/kg/min [13], via a 20-gauge cannula sited in an antecubital vein, using a syringe pump (Graseby™ 3500; Graseby Medical LTD, Watford, Hertfordshire, UK). Adenosine will be infused for a minimum of 6 minutes before acquiring stress images. Indications for terminating adenosine infusion are persistent or symptomatic third degree atrioventricular block, severe hypotension (systolic blood pressure <90 mmHg), or bronchospasm. Scans will be supervised by an MRI trained clinician with access to immediate resuscitation facilities.

##### Perfusion imaging

Patients will undergo pharmacological stress and rest perfusion. In 1.5 T scanners (Siemens Avanto, Erlangen, Germany or Intera CV release 10; Philips Medical Systems, Best, Netherlands), a breath-hold MR first-pass perfusion examination will be performed. Electrocardiographic gating and triggering will be performed using the vector cardiographic method. Fast survey images will be acquired to determine the true short axis of the left ventricle. After the acquisition of rest cine scans in the standard views, adenosine will be infused at a dose of 140 μg/kg/min for up to 6 minutes [13]. During the adenosine infusion, electrocardiographic activity will be continuously monitored, and blood pressure and heart rate measurements will be obtained every minute. Within the last minute of infusion, the adenosine stress MR perfusion scan will be performed to visualize the three short-axis slices of 8 mm section thickness using 40 dynamic acquisitions. During the inspiratory phase of the second breath, a bolus of gadodiamide (Omniscan; GE healthcare, Pittsburgh, PA**,** United States) will be injected using a power injector (Spectris; Medrad, Indianola, Pennsylvania) into an antecubital vein at a dose of 0.1 mmol per kilogram of body weight and an injection rate of 4 ml/s, followed by a 20 mL saline flush. Stress perfusion MR images will be obtained with a gradient-echo sequence by using saturation recovery steady-state free precession (SR-SSFP). After 15 to 25 minutes, identical MR perfusion scan at rest will be continued to allow adequate clearance of the first bolus of the contrast agent.

#### CMR analysis

##### Visual analysis

**Figure 3:**
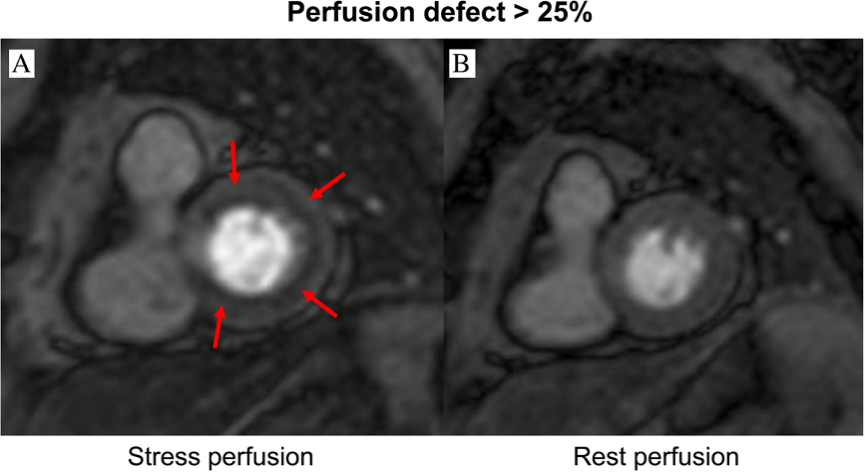
**A representative example of a patient with inducible ischemia in perfusion CMR.** **(A)** Stress perfusion **(B)** Rest perfusion. Red arrow indicates inducible ring-type ischemia in stress perfusion CMR.

Visual analysis of myocardial perfusion will be performed offline by consensus of two CMR specialists. Rest and stress perfusion images of the three short axis sections (base, mid, and apex) will be viewed side by side. The first-pass study will be analyzed both in qualitative and semi-quantitative ways, with a 16-segment left ventricle model. On qualitative assessment, perfusion defects are defined as subendocardial or transmural visually dark myocardial areas, respecting coronary distribution, with a delay of at least 2 seconds compared with remote healthy myocardium, and persisting for at least 10 frames [13]. A score will be assigned for each left ventricular segment according to the following scale: 0 = no perfusion defect; 1 = subendocardial perfusion defect with transmural extension <25%; 2 = subendocardial perfusion defect with transmural extension between >25% and 50%; 3 = subendocardial perfusion defect with transmural extension between >50% and 75%; and 4 = subendocardial perfusion defect with transmural extension >75%. For each patient a total score will be obtained as the sum of the individual scores of each segment. If a perfusion defect is also present at rest, the stress-related (ischemic) score will be calculated as the difference between the score after stress minus the score at rest. All cases will undergo evaluation for late gadolinium enhancement. A normal scan requires the absence of both reversible ischemia and late gadolinium enhancement. A representative example of a patient with inducible ischemia is shown in Figure 3.

##### Semi-quantitative analysis

Semi-quantitative analysis will be performed by signal intensity (SI)/time curves with a commercially available software (QMass MR Enterprise Solution, Advanced Edition, Medis, Leiden, the Netherlands); endocardial and epicardial borders of the three slices are traced manually, and curves and bull’s eye plots will be obtained by two independent skilled operators, with divergences being resolved by consensus.

### Echocardiography

Two-dimensional transthoracic echocardiography (TTE) will be performed at patient’s screening and 12 weeks after enrolment. TTE will be performed with a commercially available echocardiographic instrument (Vivid 7, GE Medical Systems, Milwaukee, Wisconsin, United States or Sequoia 512, Acuson, Mountain View, California, United States). A standard M-mode, two-dimensional ECG and echocardiographic Doppler study will be performed. All TTE recordings will be interpreted by staff cardiologists.

### Follow-up

All patients will visit our outpatient clinic at 1, 4, 8, 12, and 16 weeks after randomization. Each visit consists of a clinical evaluation, blood analysis, and a 12-lead ECG. After three months of udenafil treatment, adenosine-stress CMR, echocardiography, and a stress test will be performed (Table 3).

**Table 3:** **Trial process chart**

Visit	Visit 1	Visit 2	Visit 3	Visit 4	Visit 5	Visit 6	Visit 7
Status	Screening	Baseline					Follow-up phase
Week	−4 ~ 0	0	1 (±3 day)	4 (±7 day)	8 (±7 day)	12 (±7 day)	16
Informed consent	●						
Inclusion/exclusion criteria	●						
Baseline characteristics	●						
Risk factors	●						
Medical history	●						
Past medication history^1^	●						
Family history	●						
Randomization		●					
Physical examination	●					●	
Vital signs	●	●		●	●	●	
Height/weight^2^	●	●		●	●	●	
Blood analysis^3^	●					●	
Pregnancy test^4^	●					●	
ECG	●					●	
Echocardiography	●					●	
Exercise test	●					●	
CMR^5^	●					●	
CAG or CCTA	●						
SF-36 questionnaire	●					●	
BISF-W self-assessment questionnaire	●					●	
Case drug distribution		●		●	●		
Case drug collection				●	●	●	
Trial evaluation							
Adverse events	●	●	●	●	●	●	●
Patient’s compliance			●	●	●	●	
Concomitant medications^6^		●	●	●	●	●	

CMR; Cardiac magnetic resonance image, CAG; Coronary angiography, CCTA; Coronary CT angiography, ECG; Electrocardiogram.

^1^The participant’s usual medication regimen continued unchanged throughout study duration.

^2^Check weight only after Visit 2.

^3^CBC: WBC, Hemoglobin, Hematocrit, Platelet; Chemistry: Total protein, Albumin, Globulin, Total bilirubin, AST; aspartate aminotransferase, ALT; alanine transaminase, ALP; alkaline phosphatase, BUN; blood urea nitrogen, Creatinine, Uric acid; Electrolyte: Na, K, Cl; NT-proBNP; N-terminal of the prohormone brain natriuretic peptide; hs-CRP; high-sensitivity c-reactive protein.

(NT-proBNP, hs-CRP: check visit 1 and visit 5).

^4^Women of child-bearing age.

^5^Stop all medications three days before CMR.

^6^Concomitant medications: beta-blockers, calcium channel blockers, angiotensin converting enzyme inhibitors, angiotensin receptor blockers, statin, and trimetazidine.

During follow-up visits all adverse events including chest pain, headache, and so forth, will be documented. Serious adverse events will be immediately reported to the principal investigator. To ensure the quality of this trial, the clinical monitors will survey and document the trial process once a month in each participating hospital. Patient dropouts and the reasons, and patients’ compliance will be recorded at three months after randomization.

### Statistical analysis

#### Sample size calculation

We calculated the sample size for this study using a superiority categorical variable sample size formula. We assumed the improvement of perfusion defect size as >25% in adenosine-stress CMR from baseline to three months after udenafil treatment. According to the researcher’s intuition, we assumed the improvement of perfusion defect as 2% in the control group. If we applied two tails, an 80% power, an α-level of 0.05, and 10% dropout rate, we would need a total of 76 patients: 38 patients in the udenafil treatment arm and 38 patients in the control arm.

The prevalence of variant angina in Asian population is reported to be between 1 to 5% [14]. For a total of 76 patients, 4 patients with variant angina will possibly be enrolled in this study. In order to achieve scientifically robust results, we will enroll a total of 80 patients (40 patients in the udenafil treatment arm and 40 patients in the control arm).

#### Statistical analysis

For comparison between the udenafil treatment and the control group, we would use student’s t-test for continuous variables and χ^2^-test for categorical variables. To evaluate improvement of perfusion defect size >25% between udenafil treatment and control group, we would analyze using Fisher’s exact test. The difference in the continuous outcome between the experimental and control group will be assessed using generalized linear models. Frequency differences will be tested using χ^2^-test or Fisher’s exact test. We will be analyzing this data using an intention-to-treat analysis. All randomized patients will be included in the analysis. A *P* value of less than 0.05 will be considered statistically significant.

### Trial organization

#### Executive committee

The Executive Committee will be composed of the study chairperson and the principal investigators of the investigating centers. This committee will approve the final trial design and protocol issued to the Data and Safety Monitoring Board (DSMB) and the clinical sites. This committee will also be responsible for reviewing the final results, determining the methods of presentation and publication, and the selection of secondary projects and publications by members of the Steering Committee.

#### Data Safety Monitoring Board (DSMB)

An independent DSMB will be composed of cardiologists and a biostatistician following the applicable regulatory guidelines and who did not participate in the trial. The DSMB committee will review the safety data from this study and will make recommendations based on safety analyses of unanticipated device effects (UADEs), serious adverse events (SAEs), protocol deviation, and follow-up reports. In addition to the scheduled DSMB meetings (which will be determined prior to the initiation of the study) the board will convene a meeting at any time if safety problems become an issue. The DSMB are responsible for recommending to the Executive Committee to modify or stop the study if there are any safety or compliance issues. However, the final decision regarding study modifications will rest with the Executive Committee. Cumulative safety data will be reported to the DSMB and will review on an ongoing basis throughout enrollment and follow-up to ensure the safety of the patients. Every effort will be made to allow the DSMB to conduct an unbiased review of the patients’ safety information. All DSMB reports will be made available to the appropriate agencies upon request but otherwise will remain strictly confidential. Prior to the DSMB’s first review of the data, the DSMB charter will be drafted. This plan defines the stopping rules for stopping the trial for safety. The first meeting of the DSMB will be requested for discussion of the protocol and an understanding of all the protocol elements. The DSMB will develop a consensus understanding of all trial endpoints and definitions used in the event adjudication process.

#### Clinical Event Adjudication Committee (CEAC)

The Clinical Events Adjudication Committee (CEAC) is comprised of cardiologists who are not participants in the study. The CEAC is responsible for the development of specific criteria used for the categorization of clinical events and clinical endpoints in the study, which are based on protocol. At the onset of the trial, the CEAC establishes clear rules stating the minimum amount of data required and the algorithm followed in order to classify a clinical event. All members of the CEAC are blinded to the primary results of the trial and meet regularly to review and adjudicate all clinical events in which the required minimum data is available. The committee also reviews and rules on all deaths that may occur throughout the trial.

#### Data coordination and site management

Data coordination and site management services were performed by the Clinical Trials Center at Seoul National University Bundang Hospital.

#### Ethical approval

The study was approved by the institutional review board of each center (Samsung Medical Center Institutional Review Board (approval number 2011-07-048-001), Seoul National University Bundang Hospital Institutional Review Board (approval number B-1106-129-016)) and will be carried out in compliance with the Declaration of Helsinki.

## Discussion

### Treatment option of MVA

There exist several different therapeutic approaches to the treatment of MVA. The primary goal of medical therapy is to ameliorate the chest pain. However, the conventional anti-anginal treatment is often unsuccessful in MVA patients [1]. Lanza *et al*. reported that atenolol was associated with improvement in chest pain episodes, while amlodipine and nitrate were not [15, 16]. Kaski *et al*. reported that angiotensin-converting enzyme inhibition lessens exercise-induced ischemia in MVA patients [17]. Some authors define MVA as a part of visceral pain syndrome which is not related to myocardial ischemia, and reported that imipramine improved the symptoms in MVA patients, possibly through a visceral analgesic effect [18]. Taken together, there exists no standard treatment guideline for MVA.

### Rationale for the use of udenafil in MVA patients

The exact pathogenesis for MVA is unknown. However, it is of note that up to 50% of the resistance to blood flow in the epicardial coronary artery is determined by the small coronary blood vessels [19]. Therefore, small vessels have a significant effect on blood flow in the large vessels. Endothelial dysfunction, inflammation, and microcirculation abnormality are the three most commonly discussed mechanisms for the microvascular dysfunction [20]. Among them, endothelial dysfunction due to an impairment of endothelium-dependent vasodilation due to reduce NO release is the most commonly proposed mechanism for MVA [3]. NO exerts many of the biological actions via cGMP, which is rapidly degraded by cGMP PDE. Thus, PDEi has the potential to improve the endothelial function, which has been shown in animal and clinical studies [7–10].

### Rationale for the udenafil dosage in MVA patients

Udenafil (Zydena™) was developed in 1997 and, after clinical trials, the drug was approved by the Korea Food and Drug Administration (KFDA) in 2005. The commercially available and clinically used doses are 100 mg and 200 mg daily. Its half-life time is between 11 and 13 hours, and the estimated duration of action is approximately 24 hours. Thus, udenafil may be a good candidate drug for chronic use due to its favorable pharmacokinetic properties among PDE-5 inhibitors (sildenafil: T1/2 = 2 hours, duration of action = 4 to 8 hours; tadalafil: T1/2 = 17.5 hours, duration of action = 36 hours). In phase 3 clinical studies [21], the 100 mg and 200 mg doses showed similar efficacy (81 versus 88.5%). However, the side effect rate was lower with 100 mg daily dosing (flushing: 10 versus 23%, headache: 0 versus 23%, chest discomfort: 0 versus 5.4%, and so on).

### Rationale for the safety of udenafil use in MVA patients

A preclinical trial or phase 1 trial with PDE-5 inhibitors in coronary vasculature would minimize the risk of potential candidates being exposed to unnecessary risk. However, there are plenty of studies reporting the safe use of PDE-5 inhibitors in humans, including those evaluating the effect of PDE-5 inhibitors on the coronary arteries in humans[10–12]. There exist at least 11 isoforms of PDE, and the differential distribution of PDE isoforms in various tissues is the basis for the potential tissue-specific effect of a PDE inhibitor. Various tissues have different PDE isoform activities. Corpus cavernosum contains high activities of PDE isoforms 2, 3, and 5; human pulmonary circulation has PDE isoforms 1, 3, 4, and 5; and coronary arteries have PDE isoforms 1, 2, 3, 4, 5 [22]. Consequently, human coronary arteries can be a target of PDE-5 inhibitors.

In clinical trials, sildenafil contributes little to the increase in myocardial flow that occurs during exercise or reactive hyperemia [23], but coronary hemodynamics and circulation are not unfavorably altered after treatment with sildenafil and other PDE-5 inhibitors. These study results imply that PDE-5 inhibitors are safe, but less likely to be effective. Nevertheless, in patients with endothelial dysfunction, the PDE-5 inhibitors would lead to significant hemodynamic improvement. In dysfunctional endothelium, the release of NO by the endothelium is significantly compromised [24]. Halcox *et al*. [25] demonstrated that coronary arterial segments with endothelial dysfunction dilated significantly after application of acetylcholine combined with sildenafil. This finding infers that the inhibition of cGMP breakdown by PDE-5 inhibitors may, at least in part, compensate for reduced NO-related cGMP production due to endothelial dysfunction. Consequently, in patients with MVA with endothelial dysfunction the administration of PDE-5 inhibitors may significantly restore the NO-dependent vasodilation, leading to symptom improvement and improved perfusion in CMR.

Regarding safety concerns, animal studies did not show any coronary steal phenomenon caused by PDE-5 inhibitors [26, 27]. In humans, oral sildenafil produced only small decreases (<10%) in systemic arterial and pulmonary arterial pressures, and it had no effect on pulmonary-capillary wedge pressure, right atrial pressure, heart rate, or cardiac output. Most importantly, sildenafil did not alter coronary flow at rest in stenosed arteries and arteries without angiographically high-grade stenosis, but it increased coronary flow reserve to a similar extent in stenosed and non-stenosed arteries (by 13%), rejecting the possible concern of coronary steel phenomenon [28]. Halcox *et al*. [25] also showed a small beneficial effect on ST-segment depression during exercise testing in patients with known coronary artery disease. These studies demonstrate that coronary hemodynamics and circulation are not unfavorably altered after treatment with sildenafil and other PDE-5 inhibitors; indeed, coronary blood supply may experience a slight improvement. Taken together, there exists clinical data that PDE-5 inhibitors are not harmful in coronary artery patients, and although the effect of PDE-5 inhibitor is limited in normal coronary circulation, in patients with endothelial dysfunction the effect of PDE-5 inhibitors may be clinically significant.

The UMPIRE study is a randomized clinical trial to investigate the cardioprotective properties of udenafil in female MVA patients by means of measuring adenosine-stress perfusion defect size in cardiac MRI. If udenafil improves myocardial perfusion, udenafil may provide a valuable therapeutic option to reduce myocardial ischemia and improve cardiac function in female MVA patients.

### Study limitation

Cardiac syndrome X, or MVA, is generally characterized by patent coronary tree, chest discomfort (typical or atypical angina) with positive evidence of a perfusion defect. Prinzmetal angina patients also complain of chest pain with a normal coronary tree during an attack-free period. Thus, if a pharmacologic provocation test (with ergonovine or acetylcholine, for example) is not performed, there will be a risk for enrolling variant angina patients and this may jeopardize the study. Variant angina patients have distinctive clinical features that may be used to distinguish them from MVA patients. First, the chest pain character differs: while most MVA patients complain of ‘exertional chest pain’, variant angina patients complain of ‘resting chest pain’ which often occurs during early morning and is aggravated by alcohol consumption. Second, variant angina patients do not have vasospasm during an attack-free period. Thus, when performing CMR during an attack-free period, they will have a negative adenosine-stress CMR and will not be enrolled. Third, even if variant angina patients have a coronary vasospasm without chest pain, the perfusion defect in CMR will likely follow a coronary territory, since there exists a clear vessel preference in variant angina patients, mostly involving the right coronary artery. Although we may identify variant angina patients among possible MVA patients by using clinical characteristics and CMR results, we cannot completely exclude the possibility of enrolling variant angina patients. Because the prevalence of variant angina in Asian populationis reported to be between 1 and 5% [14], we will increase the sample size from 70 to 76 patients, in order to achieve scientifically robust results. Despite these measures, the possibility of enrolling patients with variant angina remains a major limitation.

## Trial status

The trial is currently in the recruitment phase. (Recruitment began on 20 November 2012, Expected to finish on November 2015).

## Abbreviations

CCTA: Coronary CT angiography
CEAC: Clinical Events Adjudication Committee
cGMP: 3’5’-Guanosine monophosphate
CMR: Cardiac magnetic resonance image
DSMB: Data and Safety Monitoring Board
ECG: Electrocardiography
MR: Magnetic resonance image
MVA: Microvascular angina
NO: Nitric oxide
PDE: Phosphodiesterase
PDEi: Phosphodiesterase-5-inhibitor
TTE: Transthoracic echocardiography
SAEs: Serious adverse events
SS-SSFP: Saturation-recovery steady-state free precession
UADEs: Unanticipated device effects
QoL: Quality of life.
